# Limited ability of increased sequencing depth in detecting cases missed by noninvasive prenatal testing: a comparative analysis of 3 clinical cases

**DOI:** 10.1038/s41598-024-52767-0

**Published:** 2024-01-27

**Authors:** Yinghong Lu, Na Zuo, Minxia Ning, Yuling Xie, Weiwu Liu, Sisi Ning, Yi Liang, Xiao Chen, Yuping Zhang, Jun Feng, Yunrong Qin

**Affiliations:** 1 Department of Clinical Laboratory, Yulin Women and Children Health Care Hospital, Yulin, 537000 Guangxi Zhuang Autonomous Region People’s Republic of China; 2 Department of Obstetrics, Yulin Women and Children Health Care Hospital, Yulin, 537000 Guangxi Zhuang Autonomous Region People’s Republic of China; 3 Department of Eugenic Genetics, Yulin Women and Children Health Care Hospital, Yulin, 537000 Guangxi Zhuang Autonomous Region People’s Republic of China; 4 Department of Child Healthcare, Yulin Women and Children Health Care Hospital, Yulin, 537000 Guangxi Zhuang Autonomous Region People’s Republic of China

**Keywords:** Genetics, Molecular biology, Health care

## Abstract

Increased sequencing depth can improve the detection rate of noninvasive prenatal testing (NIPT) for chromosome aneuploidies and copy number variations (CNVs). However, due to the technical limitations of NIPT, false-positives and false-negatives are inevitable. False-positives for aneuploidy and CNVs have been widely reported, but few missed cases have been reported. In this study, we report 3 patients missed by NIPT, which were still missed after increasing the sequencing depth. To verify the detection efficiency of the platform, the results of NIPT in 32,796 patients treated in Yulin Women and Children Health Care Hospital from 2020 to 2022 were retrospectively analyzed. Data on false-negative cases found by postnatal follow-up or amniocentesis were collected, and the sequencing data, pregnancy examination data, and postnatal follow-up results of these missed patients were summarized. Five patients missed by NIPT were found, and they were missed again by retesting or increasing the sequencing depth. Except for hypospadias found in 1 patient, ultrasonography of the other 4 patients showed no obvious abnormalities during the whole pregnancy. Our results suggest that pregnant women should be fully informed of the benefits and limitations of NIPT before undergoing the examination to avoid unnecessary medical disputes.

## Introduction

The application of NIPT in the clinical detection of fetal aneuploidy became possible due to the discovery of cell-free fetal DNA (cff-DNA) in maternal plasma and the rapid development of next-generation sequencing. Compared to the traditional serum screening test based on sonography and maternal biochemistry, which has a detection rate of 50–95% and a false-positive rate of 5%^1^, NIPT has increased specificity and sensitivity and therefore has gradually become a mainstream technology in prenatal aneuploidy screening. Studies have reported that the incidence of chromosome abnormalities in neonates is 1.70–7.30/10,000, and the incidence of microdeletions and microduplications is 0.30–6.81/10,000, resulting in an increasing trend and an overall incidence of chromosome abnormalities of 12.09–39.22/10,000^2^. As the detection range of NIPT covers the whole genome, there is an opportunity to detect both sex chromosome abnormalities and autosome abnormalities, although its positive predictive value and the negative predictive value are much lower.

A recent study of 20,626 pregnant women reported that the specificity of NIPT for trisomy 13, trisomy 18, trisomy 21, sex chromosome abnormalities, and copy number variations (CNVs) was 99.96%, 99.94%, 99.90%, 99.82%, and 99.89%, the positive predictive value (PPV) was 11.11%, 50.00%, 71.01%, 46.38%, and 39.47%, respectively, and the sensitivity was all 100%^3^. A recent study^4^ reported that the false-positive rate could be reduced by simulated confined placental mosaicism proportion (SCPMP), an optimized threshold method based on cell-free fetal DNA fraction enrichment. The overall sensitivity of cell-free DNA sequencing using the in-house CNV fraction-based detection algorithm for CNVs was 90.6%, and the sensitivities were 78.57% and 100% for sequences smaller than 3 Mb and larger than 3 Mb, respectively. For the fetal fraction, the sensitivity was 57.14% in the group with a fraction less than 10% and 100% in the group with a fraction more than 10%^5^. With the development of sequencing technology, the detection range of NIPT has been extended from aneuploidies, such as trisomy 13, 18, and 21, to subchromosomal CNVs as small as 3 Mb in size by increasing the sequencing depth^6–8^. It is widely believed that NIPT extensibility has a good detection rate for chromosome aneuploidies and deletions larger than 10 Mb^9^. Since NIPT is only a screening technique, the positive results obtained from NIPT need to be confirmed by invasive prenatal examinations such as karyotype analysis, chromosome microarray analysis (CMA), and copy number variation sequencing (CNV-Seq)^10^.

It is well known that cell-free fetal DNA detected by NIPT comes from placental trophoblast cells. False-positives or false-negatives may occur when the genetic material of placental trophoblasts is not consistent with that of the fetus, known as confined placental mosaicism (CPM)^11^. When confined placental mosaicism occurs, both NIPT and invasive diagnostic methods may fail to detect chromosome abnormalities. In this case, increasing the sequencing depth does not improve the detection rate.

Many studies on NIPT have been published, but the accuracy of the false-positive rates for different NIPT tests is still questioned, and the reported false-negative rates are most likely too low^12^. There have been many studies on false-positives and false-negatives regarding the target diseases of NIPT, but few have reported the missed detection of sex chromosome abnormalities and other CNVs. In this retrospective study, we report 5 cases of NIPT-negative results that were determined to be chromosome abnormalities by amniocentesis or postnatal tests. The original maternal blood samples for NIPT were retested by increasing the sequencing depth or the original method, the sequencing results were compared before and after, and the influence of the sequencing depth on the detection rate was summarized. At the same time, the pregnancy phenotype of similar cases easily missed by NIPT was suggested by summarizing the pregnancy examination and postpartum follow-up data of these cases, which should remind clinicians to carry out adequate informed education for pregnant women before implementing NIPT to avoid adverse pregnancy outcomes and unpleasant disputes.

## Patients and methods

### Patient recruitment and sample collection

We reviewed the NIPT results of 32,796 pregnant women treated in Yulin Women and Children Health Care Hospital from 2020 to 2022, and 5 missed patients were found. Pregnant women who underwent NIPT were informed that their medical data may be used and published in an anonymous form. Each participant underwent detailed genetic counseling and signed a written consent form. This study was approved by the medical ethics committee of the abovementioned hospital (YXLL20200416-1, and YLSFYLL2023-06-12-13), and all research was performed in accordance with relevant guidelines/regulations.

8–10 ml venous blood samples were collected for the NIPT. Fetal chromosome aneuploidy testing kits (Hangzhou Berry Gene Diagnostic Technology Co., Ltd., Hangzhou, China) were used for cfDNA extraction, library construction, quality control, and library pooling, following a previously published method^13^. Approximately 5 million raw sequencing reads were produced from the genome with a DNA sequence length of 36 base pairs, resulting in a sequencing depth of 0.06 × for NIPT^13^. RUPA extreme speed information analysis method to filtrate sequencing reads and about 3.5 million were uniquely mapped to the hg19 human reference genome^14^. Fetal DNA concentration (fetal%) was calculated utilizing both Y chromosome-based^15^ and cfDNA size-based^16^ methodologies. For aneuploid samples, the fetal% was approximated based on the disparity in genomic proportion of the abnormal chromosome between the reference samples and the subject sample^16^. By applying normalized chromosome representation (NCR) and GC correction^14^, we generated a Z-score within the standard range of − 3 to 3, which was used to evaluate the status of all 24 chromosomes. Samples that did not meet quality control criteria for GC content (outside 38–42%), unique reads mapping to the hg19 human reference genome (below 3.5 million), or fetal DNA concentration (less than 3.5%) were excluded^17^. At present, in China, NIPT is only used for trisomy 21/18/13, and other abnormalities are not in the range of target diseases. In addition, in China, NIPT is prohibited from reporting fetal sex.

### Bioinformatic analysis

The positive rate and positive predictive value of different types of additional findings in the women who underwent NIPT at Yulin Maternal and Child Health Care Hospital from 2020 to 2022 were summarized and analyzed, and clinical information of false-negative/missed patients was collected. For missed patients, the key data of primary NIPT results were summarized to ensure quality control.

### Short tandem repeat (STR) test

To exclude sample mistakes, an STR test was performed for the blood samples of the children and the samples evaluated by NIPT to preliminarily determine the relationship between them. The sample type of the child was a dry blood spot card or peripheral blood sample. According to the Public Safety Industry Standard of the People's Republic of China GA/T383-2014, DNA was extracted and amplified by PCR using the AGCU Exepressmarker 22 fluorescence detection kit (Wuxi Zhongdermeilian Biotechnology Co., LTD). Genotype analysis was performed by capillary electrophoresis and GeneMapper ID-X analysis software using the 3500DX gene analyzer (ABI).

### Sequencing depth increase

The samples missed by NIPT were reevaluated with an increased sequencing depth using the high-throughput sequencing platform Illumina, Berry Genomics, and then the results were analyzed. 20 million raw reads with identical sequence length were obtained, leading to a sequencing depth of approximately 0.24 × with NIPT PLUS. Filtrated by RUPA analysis method, about 10 million uniquely mapped reads were allocated to continuous nonoverlapping 100-kb bins. A hidden Markov model (HMM) was used to detect the CNVs. Using algorithm advances for detecting CNVs from NGS data^18^ 0, a principal component analysis (PCA) based method addressed the signal-to-noise ratio issue.

## Results

### Bioinformatic analysis

The results of 32,796 patients who underwent NIPT were statistically analyzed. A total of 535 patients with positive results beyond trisomy 13/18/21 were reported. The statistics of different types of positive results are shown in Tables 1, 2, 3 and 4. Of the 535 patients, 422 received an invasive prenatal diagnosis, and the results were consistent with the NIPT results for 162 patients, showing a PPV of 38.39%. Among the 535 patients with positive results, 193 had abnormal sex chromosomes, with a PPV of 36.02%. Among the 193 patients with sex chromosome abnormalities, the PPV was the highest for XYY (90.91%), and the PPV was lowest (0%) for maternal abnormalities. The positive predictive value was 8.0% in 34 patients with aneuploidy of other chromosomes. There were 308 patients with other CNVs, and the PPV was 42.86%. The original NIPT data of the five missed cases were reviewed and analyzed, and all the links of quality control were passed (Table 5).

**Table 1:** Analysis of distribution and positive predictive value of additional abnormal results of NIPT for aneuploidies and CNVs.

Chromosome	NIPT positive	Invasive testing	True positive	PPV (%)
1	18	16	5	31.25
2	22	18	11	61.11
3	22	17	7	41.18
4	23	18	10	55.56
5	16	13	4	30.77
6	18	17	8	47.06
7	36	27	5	18.52
8	21	16	4	25.00
9	16	15	6	40.00
10	7	5	0	0.00
11	11	9	4	44.44
12	8	8	5	62.50
13	8	7	3	42.86
14	9	5	1	20.00
15	8	5	1	20.00
16	23	13	3	23.08
17	16	12	9	75.00
18	6	5	2	40.00
19	4	4	0	0.00
20	4	3	0	0.00
21	6	4	3	75.00
22	40	24	13	54.17
X/Y	193	161	58	36.02
Total	535	422	162	38.39

*PPV* positive predictive value.

**Table 2:** Types and positive predictive values of abnormal results of sex chromosome in NIPT.

Type of SCA	NIPT positive	Invasive testing	True positive	PPV (%)
45, X	102	84	14	16.67
47, XXX	25	21	9	42.86
45, X (Mat)	2	0	0	0.00
47, XXX (Mat)	7	6	0	0.00
47, XXY	34	31	25	80.65
47, XXY (Mat)	10	8	0	0.00
47, XYY	13	11	10	90.91
Total	193	161	58	36.02

*SCA* sex chromosome aneuploidy, *Mat* maternal origin, *PPV* positive predictive value.

**Table 3:** Positive predictive value of aneuploidy in additional abnormal results of NIPT.

Chromosome	NIPT positive	Invasive testing	True positive	PPV (%)
2	1	1	1	100.00
3	1	1	0	0.00
4	1	0	0	0.00
5	1	1	0	0.00
6	1	1	0	0.00
7	17	10	0	0.00
8	3	2	0	0.00
9	2	2	1	50.00
10	1	1	0	0.00
14	1	1	0	0.00
15	1	1	0	0.00
16	1	1	0	0.00
19	1	1	0	0.00
22	2	2	0	0.00
Total	34	25	2	8

*PPV* positive predictive value.

**Table 4:** Detection and positive predictive value of NIPT for CNVs of different lengths.

Size (Mb)	NIPT positive	Invasive testing	True positive	PPV (%)
< 5	224	167	75	44.91
5–10	35	30	12	40.00
10–15	28	24	8	33.33
> 15	21	17	7	41.18
Total	308	238	102	42.86

*PPV* positive predictive value.

**Table 5:** Analysis of raw NIPT data of samples from 5 cases missed by NIPT.

cases	DNA concentration (ng/μl)	Library concentration (pM)	UniMap reads (M)	Fetal%	Q30	GC%	Gender	Result
Case 1	0.355	716.5	3.7	13.81	95.92	39.78	Male	Low risk
Case 2	0.084	112.9	2.7	9.26	95.55	40.36	Male	Low risk
Case 3	0.15	103.8	2.9	7.45	95.3	39.52	Female	Low risk
Case 4	0.13	128.8	2.8	18.27	95.2	40.32	Male	Low risk
Case 5	0.148	160.64	2.9	3.99	95.13	39.42	Female	Low risk
Reference range	0.05–0.6	> 10	> 1.5	> 4	> 80	39–42	–	–

*Q30* refers to the percentage of bases with sequencing errors less than or equal to 1/1000.

*GC%* GC content refers to the percentage of guanine (G) and cytosine (C) nucleotides in a DNA sequence.

*Fetal%* fetal fraction refers to the proportion of fetal DNA in maternal plasma during pregnancy.

### False-negative cases

#### Case 1

Patient 1 (21YL01463) was a 24-year-old woman with an NT value of 1.1 and a low-risk traditional fetal aneuploidy screening test result who chose to undergo the NIPT test voluntarily at 15^+6^ weeks of gestation. The results indicated a low risk. She reported no history of special medications or contact during pregnancy. Ultrasonography examination at 21 + weeks of gestation revealed abnormal fetal genitalia. Amniotic fluid was extracted for G-banding karyotype analysis and CNV-Seq detection, and the results showed 47, XYY and an additional 2.72 Mb duplication at 1q21.1q21.2. The gravida insisted on continuing the pregnancy. No other significant abnormalities were observed after birth.

#### Case 2

Patient 2 (21YL06201) was a 32-year-old woman who did not undergo traditional prenatal screening and chose to undergo NIPT at 18^+2^ weeks of gestation. The results indicated a low risk. Ultrasonography and other tests during pregnancy showed no abnormalities. At 32^+1^ weeks of gestation, a baby was delivered by cesarean section and admitted to the neonatology department due to premature rupture of membranes, abnormal fetal position (transverse position), premature delivery, low birth weight, and respiratory distress. The baby had mild neonatal asphyxia and a heart rate of 100 bpm. The Apgar score was 4 points (breath-1, skin color-2, reaction-1, muscle tone-2) at 1 min, 8 points (breath-1, muscle tone-1) at 5 min, and 8 points (breath-1, muscle tone-1) at 10 min. Routine examinations, including neonatal tandem mass spectrometry, showed no significant abnormalities. The patient’s TSH level was 18.01 (reference range 0–8). The result of G-banding karyotype analysis was 45, X [93]/46, XY [7].

#### Case 3

Patient 3 (21YL01208) was a 27-year-old woman who opted to undergo NIPT testing at 18^+4^ weeks of gestation due to the critical risk of trisomy 18 determined by serological screening, and the result indicated low risk. Ultrasound showed no abnormalities. The baby was hospitalized after birth for congenital heart malformations, hypoxic-ischemic encephalopathy, and neonatal hypoglycemia and underwent surgery to treat congenital heart disease. The baby’s clinical manifestations were growth retardation, neck muscle weakness for more than 4 months, the inability to raise its head, and the inability to walk independently at the age of 16 months. G-banding karyotype analysis and CNV-Seq detection were performed, and the results showed del (9) (p24.3p22.3)(15.18 Mb).

#### Case 4

Patient 4 (22YL01294) was a 30-year-old twin-pregnant woman who conceived by IVF-ET. Ultrasonography showed foot varus in one of the twins. NIPT performed at 22^+0^ weeks of gestation indicated a low risk. At 37^+6^ weeks of gestation, the twins were delivered by cesarean section due to chronic fetal distress, fetal malformation, growth restriction, and gestational diabetes. One baby weighed 2650 g, and the other weighed 2050 g. The clinical phenotype of the infant with low body weight was hypospadias, with the presence of a penis but no testicles. The result of G-banding karyotype analysis was 47, XXY. No abnormality was found in the heavier infant.

#### Case 5

Patient 5 (21YL06136) was a 21-year-old woman who underwent NIPT at 12^+3^ weeks of gestation because conventional prenatal screening suggested a risk of trisomy 18, and the result showed no significant abnormalities. The fetus was delivered vaginally at 38^+3^ weeks and had low birth weight and neonatal hypoglycemia. When the infant was approximately 6 months old, he was referred to the hospital for treatment due to malnutrition and developmental delay (poor head control at 6 months). Physical examination found special features such as a small chin, short limbs, no obvious physiological curvature of the wrist joint, broken palm lines of the left palm, and muscle hypotonia. The preliminary diagnosis was developmental retardation, hydrocephalus, and congenital heart disease. The results of CNV-Seq were dup(1) (q41q44) (29.16 Mb) and del(13) (q33.3q34) (5.70 Mb). He improved after receiving intervention training and other treatments. G-banding karyotype analysis was performed for the child’s mother, and the result was 46, XX, t(1; 13) (q41; q33).

### STR test

The results of STR detection suggested that among the 21 STR loci in the detection range, the original NIPT sample was consistent with the loci of the pregnant women, or the source of alleles of the children could be found from the genotype of the original sample, which did not rule out the child's genetic relationship with the original sample and indicated that the original sample had not been confused with other samples (Table 6).

**Table 6:** Analysis of short tandem repeats (STRs) results in 5 cases of NIPT missed samples.

Locus	Case 1	Case 2	Case 3	Case 4		Case 5
				Fetus 1	Fetus 2	
D3S1358	17	16, 17	15	19	NA	15, 16
D13S317	10	8, 11	8	8, 10	8	8
D7S820	11	12	11	8	11	11
D16S539	11	11, 12	13	9	9	9
Penta E	11	19	11	11	20	12
D2S441	11	11	12	11	11	12
TPOX	8	9	8	9, 11	11, 9	8
TH01	7	7	10	9	9	9
D2S1338	26	16	17, 23	NA	23	24
CSF1PO	11	12	12	11	11	9, 10
Penta D	10	9	11, 13	9	9	9
D10S1248	13	13	13	15	15	14
D19S433	15.2	13.2	15	13, 16.2	13	13
vWA	14, 17	19	18	17	17	18
D21S11	30.2	29	30.2	30	30	27
D18S51	14	14	19	NA	NA	23
D6S1043	11	11, 19	19	11	11	12
Amel	X, Y	X	X	X, Y	X, Y	X
D8S1179	11	13	12	13	13	15
D5S818	13	10	12, 10	7	13	11
D12S391	NA	20, 21	18	20	23	24
FGA	24	21, 22	23	24	24	24

### Increased sequencing depth

**Figure 1:**
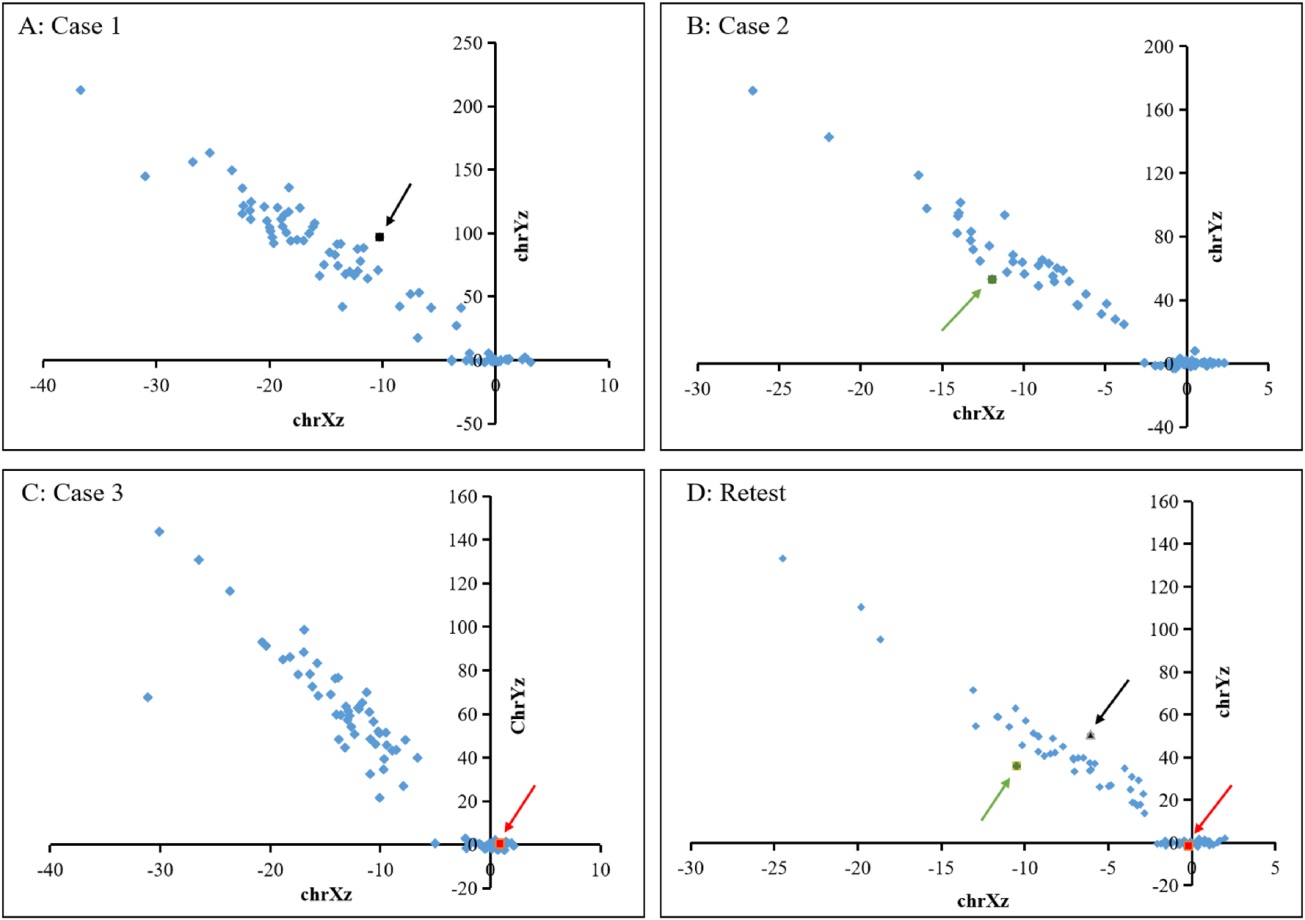
Fetal sex chromosome aneuploidy results of NIPT. (**A**) case 1 (− 9.26, 96.95). (**B**) case 2 (− 11.94, 53.15). (**C**) case 3 (0.85, 0.43). (**D**) the retest results of case 1–3 by increasing the sequencing depth. Black arrows: case 1 (− 10.49, 35.99). Green arrows: case 2 (− 6.06, 50.73). Red arrows: case 3 (− 0.18, − 1.32). Case 1 and case 2 were male, case 3 was female, and their retest results were consistent with them.

**Figure 2:**
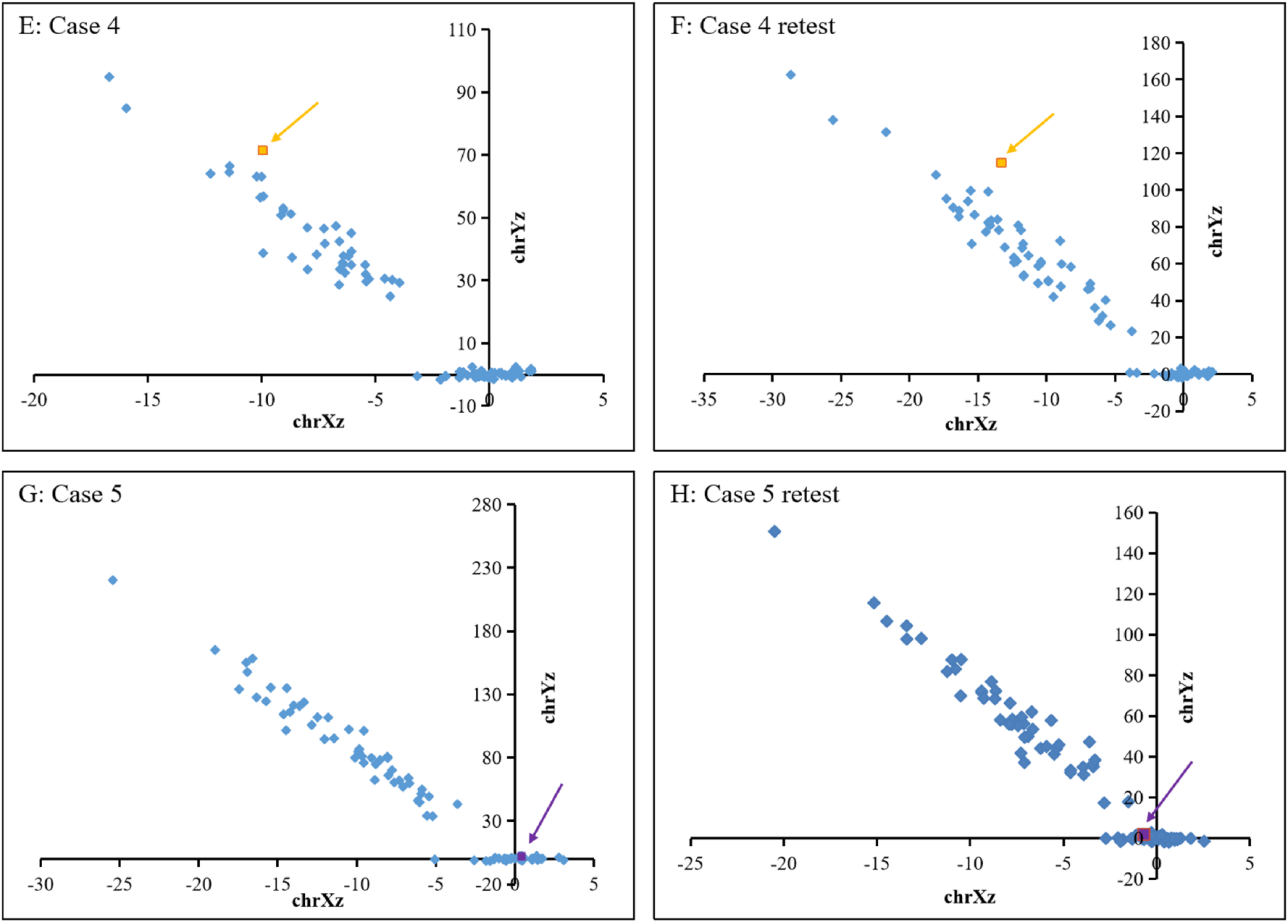
Fetal sex chromosome aneuploidy results of NIPT. (**E**) case 4 (− 9.97, 71.42). (**F**) the retest result of case 4 (− 13.29, 114.96). (**G**) case 5 (0.42, 2.07). (**H**) the retest result of case 5 (− 0.69, 1.98). Case 4 was male, case 5 was female, and their retest results were consistent with them.

The retesting results of all samples were consistent with the original results, and no abnormalities were found. The 3 missed samples (case 1–3) missed again by NIPT PLUS, with all the links of quality control were passed (Table 7). The sex chromosome test results of the 5 samples were consistent with those before retesting (Figs. 1, 2). Due to insufficient remaining available samples, NIPT PLUS retesting was not performed in Cases 4 and 5, and NIPT was used instead. However, we believe that it is more beneficial to supplement the NIPT PLUS test for these two samples.

**Table 7:** Data analysis of reexamination results of 5 NIPT missed samples.

Cases	Redetect method	DNA concentration (ng/μl)	Library concentration (pM)	UniMap reads (M)	Fetal%	Q30	GC%	Gender	Result
Case 1	Increase depth	0.245	395.3	17.9	11.99	94.72	40.17	Male	Low risk
Case 2	Increase depth	0.072	128.2	14.8	8.51	94.66	40.56	Male	Low risk
Case 3	Increase depth	0.097	156.54	14.2	7.06	94.61	40.33	Female	Low risk
Case 4	Original depth	0.152	140.95	4.5	20.09	94.3	40.37	Male	Low risk
Case 5	Original depth	0.156	170.09	4.2	5.93	95.38	39.42	Female	Low risk

Original depth: 0.06x; Increase depth: 0.24x.

*Q30* refers to the percentage of bases with sequencing errors less than or equal to 1/1000.

*GC%* GC content refers to the percentage of guanine (G) and cytosine (C) nucleotides in a DNA sequence.

*Fetal%* fetal fraction refers to the proportion of fetal DNA in maternal plasma during pregnancy.

## Discussion

Due to clinical requirements, the application of NIPT has expanded from trisomy 13/18/21 syndrome to other chromosomal aneuploidies and even CNVs. It has been shown that incorporating microaberration detection into genome-wide NIPT as part of a screening/diagnostic procedure is possible in the case of no or only a slight increase in the sequencing depth depending on the specific parameters and the purpose of the test when the variant length is above 3 Mb and the fetal fraction is above 10%, making it a potential diagnostic tool^19^. Another systematic review reported that the sensitivity of NIPT for CNV detection ranged from 20 to 100%, the specificity ranged from 81.62 to 100%, and the PPV ranged from 3 to 100%^20^. In the majority of patients with negative results screened by NIPT, confirmatory analysis was not available, and thus, the NPV could not be determined^20^. Therefore, NIPT should be used with caution for CNVs^20^. In our study, we detected additional abnormal results across all 23 pairs of chromosomes from aneuploidy to CNVs as small as less than 5Mb by NIPT without increasing the sequencing depth (Table 1), with a positive predictive rate of 42.86% for CNVs and 36.02% for sex chromosome abnormalities, although the predictive rate was low (8.0%) for autosomal aneuploidy (Tables 2, 3 and 4). These abnormalities cannot be detected in traditional serological prenatal screening, and some small segments of CNVs (usually < 10 Mb) cannot be detected by G-banding karyotype analysis alone. Therefore, although five missed patients were found in this study, NIPT is still an effective screening method for chromosome abnormalities.

In recent years, NIPT with an increased sequence depth, known as NIPT PLUS, was created to improve detection rates. As a result, the positive CNV detection rate of NIPT has increased, and the detection rate of small fragments at 25 Mb is even higher than that of karyotype analysis^21^. A previous study^22^ reported that the PPV of expanded NIPT was 100% for T21, T18, and XXY and 42.8% and 16.7% for CNVs above 10 Mb and 5–10 Mb, respectively. Another large sample study^9^ reported that the detection rate increased by 1.02% for NIPT PLUS compared with NIPT alone, and the total PPV of NIPT PLUS was 43.61%, which was higher than that of NIPT alone (12.56%), especially in cases with a CNV > 10 Mb. Increasing the sequencing depth increases not only the detection rate of CNV but also the PPV.

However, in our study, there were 5 NIPT missed samples. Cases 4 and 5 were retested by NIPT, while case 1–3 were tested by NIPT PLUS to verify the accuracy of the initial NIPT results and observe the effect of increasing the sequencing depth. Just as the results of NIPT retest were still negative in case 4–5, the results of NIPT PLUS retest in case 1–3 were also negative. The chromosome abnormalities in these five children were 47, XXY, dup(1) (q21.1q21.2)2.72Mb, del(9) (p24.3p22.3)15.18Mb, 47, XXY, dup(1) (q41q44) 29.16Mb, del(13) (q33.3q34) 5.7Mb (Table 8). After increasing the depth of sequencing, case 1–3 were still missed, with all quality control data meeting the standard (Tables 5, 7), and STR also ruled out the possibility of sample error (Table 6). That is, the ability of an increased sequencing depth in detecting samples missed by NIPT was limited, as the detection principle of both NIPT and NIPT PLUS is to detect the cell-free DNA of placental trophoblasts, which is not always consistent with true fetal DNA. When the genetic material of placental trophoblasts is inconsistent with that of the fetus, a false-positive or false-negative result will occur. In Van’s study of 404 fetuses with trisomy 21, 3.7% had normal or low-mosaic (< 30%) karyotypes, which would have been potentially missed by NIPT based on biological grounds. Similarly, it is inevitable that other chromosomal abnormalities will be missed on the same biological grounds^11^. Short-term cultured villi (STC-villi), as well as long-term cultured villi (LTC-villi), are the gold standard for the cytogenetic analysis of chorionic villi samples. The origin of the cells in STC-villi and LTC-villi is derived from the outer cell layer of CV, the cytotrophoblast, and the mesenchymal core of the inner cell layer, respectively. Only cytotrophoblast DNA is investigated in NIPT, so the results would be comparable to those of STC-villi^11,23^.

**Table 8:** Clinical information of 5 cases missed by NIPT.

Cases	Case 1	Case 2	Case 3	Case 4		Case 5
				Fetus 1	Fetus 2	
Age (years)	24	32	27	30		21
Clinical diagnosis	Not specified	Not specified	18-trisomal critical risk	Twin pregnancy, IVF-ET		18-trisomal critical risk
Gestational age (weeks)	15^+6^	18^+2^	18^+4^	22^+^		12^+3^
Serological screening	Low risk	–	18-trisomal critical risk	Low risk		18-trisomal critical risk
Weight (kg)	55	70	57	50.5		70
NT value (mm)	1.1	2.1	1.6	NA	NA	1.4
Ultrasound examination results	Suspicious abnormal morphology of fetal external genitalia	NT 2.1	Normal	Normal	Both feet pronate	Normal
Gestational age at birth (weeks)	37^+6^	32^+1^	39^+0^	37^+6^	37^+6^	38^+3^
Birth weight (g)	2600	1950	3260	2650	2050	2140
Pregnancy outcome	47, XYY, dup(1) (q21.1q21.2) (2.72Mb)	45, X [93]/46, XY [7]	del (9) (p24.3p22.3) (15.18Mb)	Normal	47, XXY	dup (1) (q41q44) (29.16Mb), del (13) (q33.3q34) (5.70Mb)
Clinical phenotype	No clinical symptoms	Respiratory distress	16-month-old still can’t walk independently, can’t speak, developmental delay	Normal	Hypospadias	Developmental retardation, hydrocephalus and congenital heart disease

*NA* not available, *IVF-ET* in vitro fertilization and embryo transfer, *NT* nuchal translucency thickness.

With increasing age, the probability of fetal aneuploid abnormalities increases, while chromosome structural abnormalities and mosaicism mostly occur in pregnant women under 40 years of age, and the rates do not increase with age. NIPT may miss 12.4% of abnormal results that should be originally found by karyotype analysis^24^. Our cases likely had CPM, which led to missed results. Unfortunately, we failed to collect placental tissue samples for verification. In addition, there is evidence that CPM is somewhat associated with adverse perinatal outcomes, and the risk of preeclampsia and low birth weight are significantly increased in these patients compared with the general obstetric population. Due to chromosomal mosaics, normal and abnormal cells are unevenly distributed across different compartments (fetal, embryonic ectoderm, and trophoblast)^25–27^, which might explain the mosaicism in case 2 in our series.

NIPT has been used in the prenatal detection of twins for many years, and its detection ability has also been clinically recognized to some extent. In Japan, more women with twin pregnancies tend to choose to undergo NIPT, especially those who conceive through assisted reproductive technology (ART)^28^. Fosler et al.^29^ reported the detection of trisomy 21 by cell-free fetal DNA (cff-DNA)-based whole-genome sequencing (WGS) NIPT in twin pregnancies and found that its performance was almost the same as that in singleton pregnancies, with a very low overall false-positive rate. However, it is known that the detection rate of NIPT for sex chromosome abnormalities is significantly lower than that for trisomy 21. In this study, case 4 involved a twin pregnancy, and the NIPT result suggested that the fetuses were male. As a result, one fetus was male, and the other was XXY. After retesting the sample by the original method, the result still indicated that the fetus was a normal male (Fig. 2). Case 1 and Case 2 were both singleton pregnancies, and the NIPT test indicated that the fetuses were male and female, respectively. The blood sample was tested after birth, and the results were XYY and 45, X [93]/46, XY [7], respectively. After increasing the sequencing depth, the results remained consistent (Fig. 1). The retest results were consistent with the original results, and the other quality control data met the standard (Tables 5, 7). The testing process of these two samples met the requirements, and there were no faults. In addition, the STR results also ruled out the possibility of sample error (Table 6). Therefore, NIPT failed to detect the sex chromosome abnormalities in cases 1, 2 and 4, which might also be caused by the limitations of NIPT technology.

Another study^30^ also reported 3 cases of sex inconsistency between NIPT and ultrasound results, and they suggested further examination and invasive prenatal diagnostic tests in cases with inconsistent results in sex assessment between NIPT and ultrasound. However, in China, sex is not allowed to be determined by NIPT, and therefore, it is impossible to obtain information about sex inconsistency between NIPT and ultrasound examination before birth. Since fetuses with sex chromosome abnormalities may have no obvious phenotype on ultrasound and may even be almost asymptomatic after birth until detection in adulthood when problems such as fertility are encountered, the utility of follow-up results after birth is limited. In addition, NIPT has only been applied to clinical use for just over a decade; therefore, false-negatives results for sex chromosome abnormalities are difficult to determine through follow-up visits. This may also be the reason why we rarely found cases of sex chromosome abnormalities during prenatal follow-up visits.

It has been reported that advanced maternal age and a decreased cell-free fetal DNA concentration are suggested to be independent risk factors for NIPT failure, and the abnormal pregnancy rate was significantly different between the successful and failed groups tested by NIPT^31^. Some studies^32^ reported three inflection points of cff-DNA at the 10th, 19th, and 30th weeks of gestation, and the concentration of cff-DNA reached the NIPT requirement after 9 weeks of gestational age; from the 10th week of gestation on, nearly 92.00% of the maternal plasma had a cff-DNA concentration > 4%. An overview of the original NIPT and baseline data of the pregnant women in this study showed that the indicators of the five missed samples met the quality control requirements: a cff-DNA concentration > 4%, maternal age between 21 and 32 years, and a gestational age between 12^+^ and 18^+^ weeks; however, they were still missed (Tables 8, 6).

When analyzing the reasons for missed samples, in addition to the analysis of the original data and the retesting of the original sample, the accuracy of the test sample should also be verified; that is, the test sample does come from the mother of the child and not another pregnant woman. Short tandem repeats (STRs) are currently the most widely used genetic markers for paternity testing^33,34^. STR verification was carried out for the 5 missed samples, and the results indicated that the original sample was indeed from the mother and was not replaced with other samples. Therefore, for samples in doubt, STR verification can be conducted. In this study, we used an STR test to verify the relationship between the mothers and children and confirmed that the samples tested by NIPT were indeed from the pregnant women themselves, thus avoiding unnecessary disputes. Therefore, when NIPT is performed, it is necessary for the laboratory to retain sufficient samples for future retesting and STR testing.

Reviewing the five patients who were missed by NIPT in this study, no special abnormality was found in other cases except Case 1, in which the fetus was suspected to have an abnormal sexual organs by an ultrasound examination before birth. In pregnant women of normal childbearing age with no ultrasound abnormalities whose traditional serological prenatal screening suggests a high risk of trisomy 18 or normal, there is no mandatory requirement for interventional prenatal diagnosis. At this time, NIPT seems to be a more reasonable choice. Unfortunately, the babies in these five cases had chromosome abnormalities. Therefore, it is necessary to define clear indications for pregnant women to choose interventional prenatal diagnosis instead of NIPT.

Even though the performence of NIPT/NIPT PLUS for testing additional abnormal results have reached a relatively ideal state, people have different attitudes regarding whether these abnormal results should be reported. These differences lie in the sociocultural context and what other principles are evoked and how these principles are defined and weighed^35^. A survey found that more than 80 percent of health care professionals in China engaging in prenatal diagnosis expressed support for extending NIPT to diseases other than common trisomies. The degree of knowledge was negatively correlated with the support rate^36^. Prenatal screening for sex chromosome aneuploidies raises complex ethical issues for future children, prospective parents, and clinicians^37^. Some participants completely disapprove of the use of NIPT for fetal whole-genome sequencing because they believe it would cause anxiety for the parents as well as their future children^38^.

Since there is no uniform standard, it is difficult to agree on the reporting principles of additional NIPT findings. Therefore, the statistical data of various testing institutions may differ. In China, when NIPT reports additional findings, pregnant women typically opt for fetal chromosomal karyotype analysis through amniocentesis rather than excluding the possibility of maternal origin. Limited by maternal consent preferences, no further confirmation of maternal karyotype via other technical means was performed on mothers with maternal abnormalities indicated by NIPT in this research.

No genetic testing of placental trophoblasts was performed in this study because chromosomal abnormalities were not detected until after birth when the placenta was no longer available or because the pregnant woman refused to provide the placenta. It is still a major challenge to verify the CPM in false-negative samples. Another limitation of this study is that only 5 cases were studied, and testing with a larger sample size is necessary. Since these five cases were identified based on clinical presentation later in pregnancy or after birth, it is possible that additional false-negative cases exist but did not present with clinical symptoms leading to confirmatory genetic testing. Therefore, the sample size of these missed cases was very small. Therefore, extending the time and the range of diseases in follow-up are conducive to the discovery of cases missed by NIPT.

## Conclusion

Generally, as a noninvasive prenatal screening method, NIPT can detect not only common trisomy 13, 18, and 21 but also other autosomal aneuploidies, deletions, or duplications of sufficiently large chromosome fragments, although the accuracy is relatively low. Increased sequencing depth may improve the overall detection rate of abnormal chromosomes, but theoretically, its detection performance is similar to that of conventional NIPT in cases of placental mosaicism. In other words, there is still a risk of false positivity or missed detection. Therefore, although noninvasive prenatal screening is popular among pregnant women, attending doctors need to fully inform pregnant women of the limitations and risks of this test to avoid unnecessary medical disputes.

## Data Availability

The datasets and material used or analysed during the current study are available from the corresponding author upon reasonable request.
